# Increased invasion and tumorigenicity capacity of CD44^+^/CD24^-^ breast cancer MCF7 cells *in vitro* and in nude mice

**DOI:** 10.1186/1475-2867-13-62

**Published:** 2013-06-24

**Authors:** Wenxing Yan, Yubing Chen, Yueliang Yao, Hongmei Zhang, Tiejun Wang

**Affiliations:** 1 Department of Radiotherapy, Second Affiliated Hospital of Jilin University, 130021, Changchun, China; 2 China-Japan Union Hospital of Jilin University, Changchun, China

**Keywords:** Breast cancer stem cells, Ultrastructure, Tumorigenicity, Invasive ability

## Abstract

**Background:**

Identification of cancer stem cells (CSCs) and their behaviors will provide insightful information for the future control of human cancers. This study investigated CD44 and CD24 cell surface markers as breast cancer CSC markers *in vitro* and *in vivo*.

**Methods:**

Flow cytometry with CD44 and CD24 markers was used to sort breast cancer MCF7 cells for scanning electron microscopy (SEM), tumor cell invasion assay, and nude mouse xenograft assay.

**Results:**

Flow cytometry assay using CD44 and CD24 markers sorted MCF7 cells into four subsets, i.e., CD44^+^/CD24^-/low^, CD44^-^/CD24^+^, CD44^+^/CD24^+^, and CD44^-^/CD24^-^. The SEM data showed that there were many protrusions on the surface of CD44^+^/CD24^-/low^ cells. CD44^+^/CD24^-/low^ cells had many microvilli and pseudopodia. The CD44^+^/CD24^-/low^ cells had a higher migration and invasion abilities than that of the other three subsets of the cells. The *in vivo* tumor formation assay revealed that CD44^+^/CD24^-^ cells had the highest tumorigenic capacity compared to the other three subsets.

**Conclusion:**

CD44 and CD24 could be useful markers for identification of breast CSCs because CD44^+^/CD24^-/low^ cells had unique surface ultrastructures and the highest tumorigenicity and invasive abilities.

## Background

Cancer stem cells (CSCs) were originally described in hematologic malignancies and have the exclusive ability to self-renew and differentiate into heterogeneous lineages of tumor cells [1,2]. The concept of CSC theory may change our perspective with regard to cancer development, progression, and treatment. CSCs are defined as a small number of tumor cells that have the ability to generate daughter tumor cells, self-renew, and maintain tumor phenotypes. In addition, CSCs are responsible for tumor resistance to chemo- and radiation therapy, recurrence, and metastasis [3-6]. Currently, searching for CSC markers is a very hot topic and some interesting data have been generated. For example, markers for CSCs of the breast, prostate, colon, and pancreas have been reported [7-9], but more work needs to be done. Furthermore, it is evident that the distinguished ultrastructure of cancer cells is linked to the tumorigenic potential for making tumor diagnoses and predicting tumor behaviors (such as recurrence, metastasis, and so on) [10-12]; however, it is unknown whether CSCs possess their own particular ultrastructure partners. Therefore, identification of CSCs and understanding of their behaviors in human cancer will provide us useful information for defining the molecular mechanisms of cancer development and progression and developing novel treatment strategies.

In human breast cancer, it has been shown that breast CSCs have a CD44^+^/CD24^-^ phenotype [2]. Consistent with this finding, knockdown of CD44 expression has been found to induce breast CSCs to differentiate into regular tumor cells without the capacity to self-renew (defined as non-CSCs) [13]. Nevertheless, in certain breast cancer patients, CD44^+^/CD24^-^ cells are still sensitive to radiotherapy, suggesting that not all breast CSCs are radioresistant [14] and that not all CD44^+^/CD24^-^ cells are CSCs. Indeed, clinical data have indicated that there is no significant correlation between CD44^+^/CD24^-/low^ tumor cell prevalence and tumor progression. The prevalence of a CD44^+^/CD24^-/low^ tumor cell did not correlate with the event-free and overall survival and did not affect the tumor response to different treatment modalities [15,16]. Thus, further investigation is needed to clarify these two markers for breast CSCs. In this study, we first sorted breast cancer cells with these two markers and then analyzed the ultrastructure of the breast CSC surface in CD44^+^/CD24^-/low^ genotypes. Furthermore, we systemically analyzed the ultrasturcture, invasion capacity, and tumor formation in breast cancer MCF7 cells with different CD44/CD24 genotypes. Our findings indicated that CD44^+^/CD24^-/low^ MCF7 cells with high numbers of microvilli and pseudopodia have greater tumorigenicity rates and invasive ability.

## Materials and methods

### Cell line and culture

The human breast cancer cell line MCF7 was obtained from the Shanghai Cell Bank (Shanghai, China) and maintained in Dulbecco’s modified Eagle’s medium (DMEM) with high glucose and supplemented with 10% fetal calf serum (FCS), 100 units/ml penicillin, and 100 μg/ml streptomycin at 37°C in 5% CO_2_.

### Flow cytometry

To sort tumor cells with CD44 and CD24 markers, MCF7 cells were seeded and grown for several days, then washed with phosphate-buffered saline (PBS), and finally harvested with 0.05% trypsin/0.025% EDTA. The cells were then detached and washed with PBS containing 2% FCS before being subjected to antibody binding, i.e., combinations of fluorochrome-conjugated monoclonal antibodies against human CD44 (FITC) and CD24 (PE) or their respective isotype controls were added to the cell suspensions at a concentration recommended by the manufacturer and incubated on ice in the dark for 20 min. After that, cells were washed twice with PBS/2% FCS and resuspended in 0.5 ml (per million cells) of PBS/2% FBS for flow cytometric analysis using a FACSVantage instrument. Anti-human CD44 FITC (Clone: IM7; 11-0441-82) and anti-human CD24 PE (Clone: eBioSN3; 12-0247-42) antibodies were obtained from Affymetrix eBioscience (http://www.affymetrix.com).

### Scanning electron microscopy (SEM)

To detect the ultrastructure of CD44^+^/CD24^-/low^ breast CSCs, we performed SEM experiments by directly using flow cytometry-sorted cells. Briefly, flow cytometry-sorted cells were seeded onto glass cover slips and fixed *in situ* with 2.5% paraformaldehyde in PBS for 2 h at 4°C. Then, the cells were rinsed in PBS and postfixed with 1% osmium tetroxide in PBS for 1 h, dehydrated through a graded ethanol series, stained *en bloc* during dehydration with a saturated solution of uranyl acetate in 70% ethanol, and then embedded in Araldite for the ultrastructural study of cells using SEM.

### Tumor cell invasion assay

Breast cancer MCF7 cells were cultured in complete cell medium and then stained with anti-CD44-FITC and anti-CD24-PE antibodies (Miltenyi Biotec, Germany). After that, the cells were washed twice with cold PBS and then stained with DAPI before being washed twice with PBS. Next, these cells were first cultured in serum-free DMEM overnight. The next day, the cells were detached with trypsin, counted, and added into the top chambers of Transwell inserts with an 8-μm pore size filter coated with Matrigel in 24-well plates. In the bottom of the chambers, DMEM containing 20% FCS was added, and the cells were then cultured for 24 h. At the end of the experiments, the cells on the top surface of the filter were removed by using a cotton-swab, and the cells on the bottom of the filter were fixed with methyl alcohol and then reviewed and quantified by a laser scanning confocal microscope (FV-1000; Olympus Japan).

### Animal experiments

Flow cytometry-sorted cells were collected, washed in PBS, and then injected into the mammary fat pad of 5-week-old severe combined immunodeficient (SCID) mice. Mice were maintained in laminar flow rooms under constant temperature and humidity and received estradiol supplementation (0.4 mg/kg) every week after cell injection. Mice were inspected for tumor appearance daily by observation and palpation for 12 weeks after cell injection. At the end of the experiments, all mice were sacrificed by cervical dislocation, and the presence of each tumor nodule was confirmed by necropsy. Experimental protocols were approved by the Ethics Committee for Animal Experimentation of the institute.

### Statistical analyses

All the in vitro experiments were repeated six times, and had similar results. Statistical analysis was performed using SPSS software version 17.0. Statistical significance was tested by using a two-tailed Student’s t-test. P<0.05 was considered statistically significant.

## Results

### Sorting breast cancer MCF7 cells using CD44 and CD24 markers

**Figure 1:**
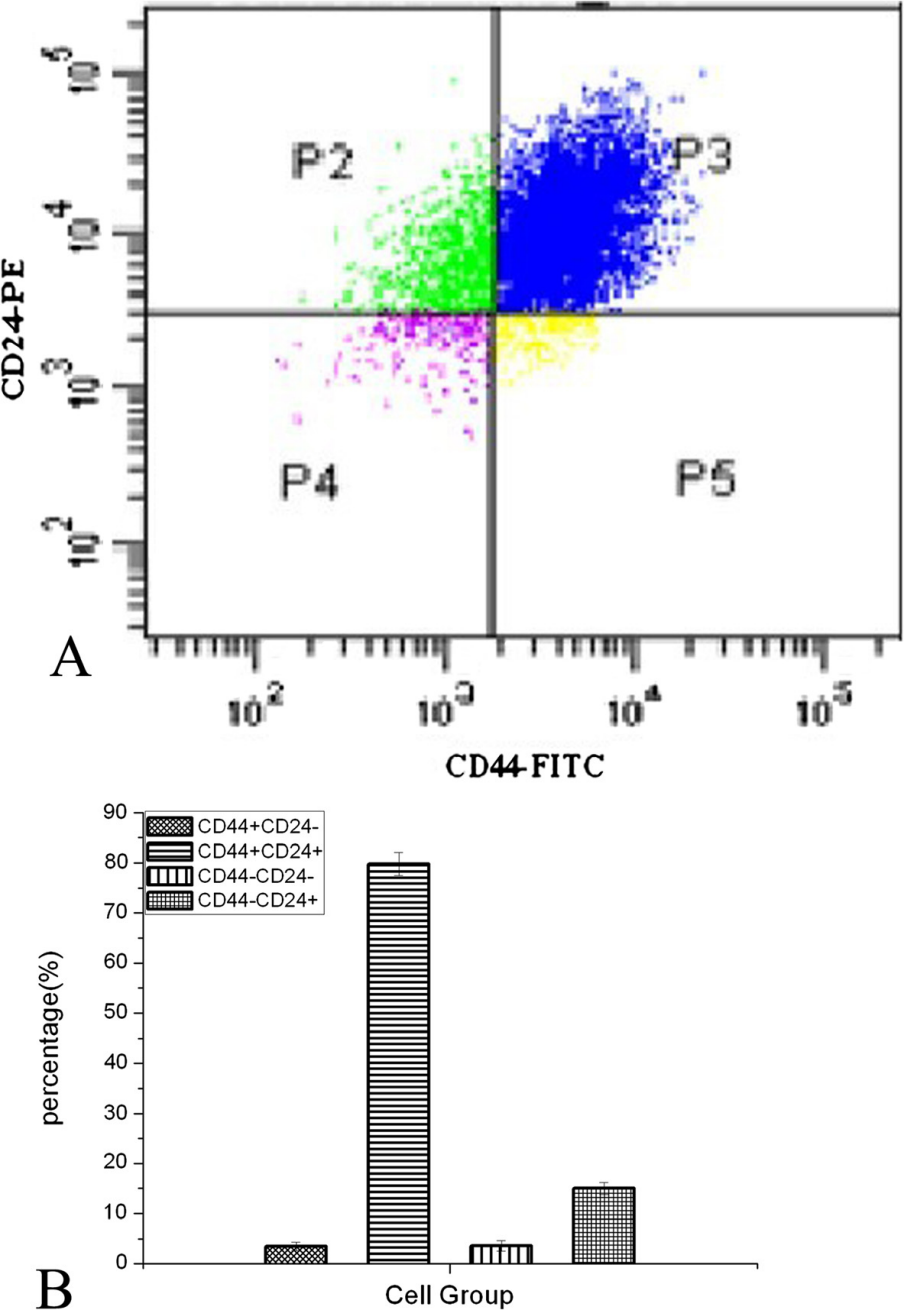
**Flow cytometry sorting of MCF7 cells using CD44 and CD24 markers. A**, MCF7 cells were analyzed by fluorescence-activated cell sorting (FACS) using anti-CD44 and anti-CD24 antibodies. **B**, Quantification of the four subsets of MCF7 cells. **C**, Confirmation of the purity of the CD44^+^/CD24^-/low^ cell type using flow cytometry.

To obtain putative breast CSCs from MCF7 cell line, we performed flow cytometry assay using antibodies against CD44 and CD24 cell surface markers. We obtained four subtypes of tumor cells (i.e., CD44^+^/CD24^-/low^, CD44^+^/CD24^+^, CD44^-^/CD24^+^, and CD44^-^/CD24^-^; Figure 1). The percentages of CD44^+^/CD24^-/low^, CD44^+^/CD24^+^, CD44^-^/CD24^+^, and CD44^-^/CD24^-^ cells were 3.5%, 79.8%, 15.0%, and 3.6%, respectively (Figure 1). According to the literature, the CD44^+/^CD24^-/low^ cells were breast CSCs (approximately 3.5%; Figure 1). The purity of this sorted cell type was further confirmed using flow cytometry. As shown in Figure 1C, the purity of the CD44^+^/CD24^-/low^ cell type was more than 90%.

### Detection of the cell surface ultrastructure of flow cytometry-sorted MCF7 cells

**Figure 2:**
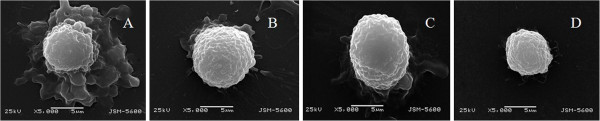
**Cell surface ultrastructure of the four different subsets of MCF7 cells. A**, CD44^+^/CD24^-/low^; **B**, CD44^+^/CD24^+^; **C**, CD44^-^/CD24^+^; and **D**, CD44^-^/CD24^-^.

To characterize the ultrastructural features of flow cytometry-sorted MCF7 cells, we performed SEM experiments. Our data showed that there are numerous protrusions on the surface of CD44^+^/CD24^-/low^ cells (Figure 2A). These cells also showed many microvilli and pseudopodia compared to the other three cell subsets (Figure 2). In contrast, CD44^+^/CD24^+^ cells showed a rough surface with several but less abundant protrusions and fewer pseudopodia on the surface compared to CD44^+^/CD24^-/low^ cells (Figure 2B). Again, CD44^-^/CD24^+^ cells showed a smooth surface with fewer protrusions and pseudopodia (Figure 2C), while CD44^-^/CD24^-^ cells showed a smooth surface with fewer protrusions and no pseudopodia (Figure 2D). These data indicate that the cell surface ultrastructure is quite different among these four subtypes of MCF7 cells.

### Invasion capacity of breast cancer MCF7 cells with different expressions of CD44 and CD24 markers

**Figure 3:**
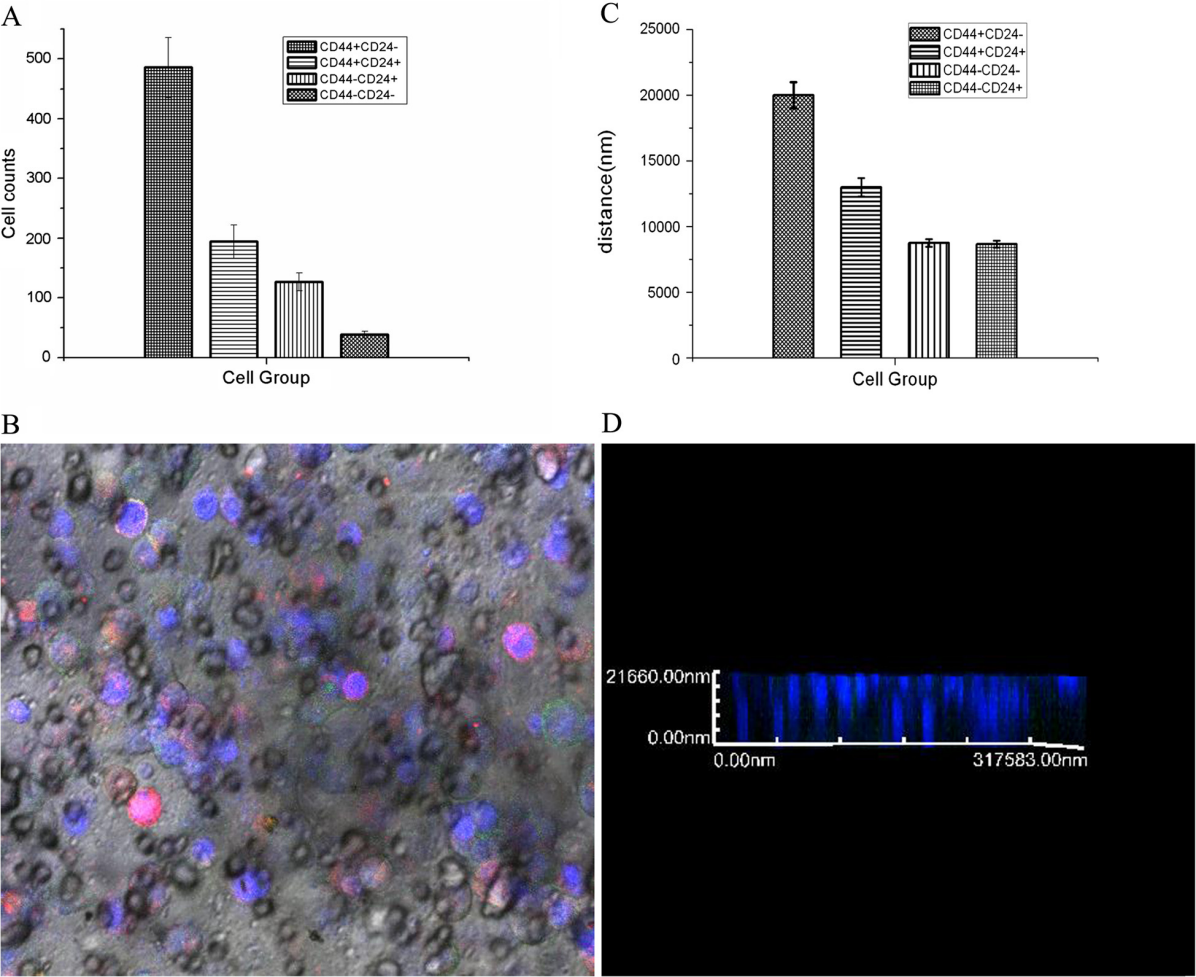
**Invasion assay of these four subsets of MCF7 cells. A**, Cell number quantification of invasion. **B**, Confocal images of these four subsets of MCF7 cells (Green: CD44^+^/CD24^-/low^; Red: CD44^-^/CD24^+^; Yellow: CD44^+^/CD24^+^; and Blue: CD44^-^/CD24^-^). **C**, Quantification of tumor cell migration distance. **D**, Images of cell migration distance.

To examine the invasion capacity of these four different subsets of MCF7 cells, we first labeled them with different colors (Green: CD44^+^/CD24^-/low^; Red: CD44^-^/CD24^+^, Yellow: CD44^+^/CD24^+^; and Blue: CD44^-^/CD24^-^). A laser confocal microscope randomly selected five fields (×400) to count the number of these four subsets of cells that invaded through the basement membrane (Figure 3). The numbers of CD44^+^/CD24^-/low^, CD44^+^/CD24^+^, CD44^-^/CD24^+^, and CD44^-^/CD24^-^ cells that invaded through the basement membrane were 485.7 ± 43.628, 194.4 ± 27.778, 126.7 ± 13.333, and 38.0 ± 3.189, respectively (Figure 3A). The number of CD44^+^/CD24^-/low^ cells that invaded through the basement membrane was significantly higher than that of the other three subsets (Figure 3B). The migration distances of CD44^+^/CD24^-/low^, CD44^+^/CD24^+^, CD44^-^/CD24^+^, and CD44^-^/CD24^-^ cells were 20011.0 ± 999.476 nm, 12996.3 ± 699.028 nm, 8664.5 ± 157.124 nm, and 8764.6 ± 99.620 nm, respectively (Table 1, Figure 3C, D). Thus, the migration distance of CD44^+^/CD24^-/low^ was significantly longer than that of the other three subsets (Table 2, Figure 3C, D). These results suggest that CD44^+^/CD24^-/low^ cells have the highest invasion capacity compared to the other three subsets of MCF7 cells.

**Table 1:** Invasion capacity of these different MCF7 subtypes

**Cell subtypes**	**Invasion cell number**	**n**	**t**	***p***
CD44^-^/CD24^+^	126.7 ± 13.33	6	5.77	0.004
CD44^+^/CD24^+^	38.0 ± 3.18	6	20.69	0.000
CD44^-^/CD24^-^	194.4 ± 27.77	6	4.21	0.013
CD44^+^/CD24^-^	485.7 ± 43.62	6	—	—

**Table 2:** Migration distances of these different MCF7 subtypes

**Cell subtypes**	**Migration distances**	**n**	**t**	***p***
CD44^-^/CD24^+^	8664.3 ± 157.12	6	−19.45	0.000
CD44^+^/CD24^+^	12996.2 ± 699.02	6	7.25	0.002
CD44^-^/CD24^-^	8764.0 ± 99.62	6	−9.80	0.001—
CD44^+^/CD24^-^	20011.0 ± 999.47	6	—	

### Xenograft formation and growth capacity among these four subtypes of CD44 and CD24-expressing breast cancer cells in nude mice

**Figure 4:**
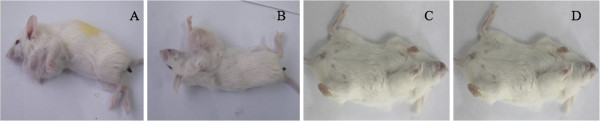
***In vivo *tumor xenograft assay of these four subsets MCF7 cells. A**, CD44^+^/CD24^-/low^; **B**, CD44^+^/CD24^+^; **C**, CD44^-^/CD24^+^; and **D**, CD44^-^/CD24^-^.

To compare the tumor-initiating capability of different MCF7 cell subsets, we injected the four flow cytometry-sorted cell subsets at different cell numbers (1×10^3^, 1×10^4^, and 1×10^5^) into the mammary left fat pad of nonobese diabetic (NOD)/SCID mice. All mice were observed for 3 months for tumor formation and growth (Table 3 and Figure 4). Briefly, 13 days after the injection of 1×10^3^ CD44^+^/CD24^-/low^ cells, a palpable tumor formed in one of six mice. Meanwhile, after the injection of 1×10^4^ CD44^+^/CD24^-/low^ cells, tumors formed in four of six mice at 11 ± 1.2 days. Furthermore, an injection of 1×10^5^ CD44^+^/CD24^-/low^ cells formed tumors in all six mice at 9 ± 2.3 days. In contrast, of the other three subsets of MCF7 cells, even with an injection of 1×10^5^ cells, only CD44^+^/CD24^+^ cells formed tumors in two of six mice at day 21 and none of the CD44^-^/CD24^-^ and CD44^-^/CD24^+^ cells were able to form tumors. These data suggest that CD44^+^/CD24^-^ cells have the highest tumorigenic capacity compared to the other three cell subsets.

**Table 3:** ***In vivo *tumor xenograft assay of these four subsets of MCF7 cells**

**MCF7 cell subset**	**Tumor formation with the indicated number of cells**		
	**1×10**^**3**^	**1×10**^**4**^	**1×10**^**5**^
CD44^+^/CD24^-/low^	1/6	4/6	6/6
CD44^-^/CD24^+^	0/6	0/6	0/6
CD44^+^/CD24^+^	0/6	0/6	2/6
CD44^-^/CD24^-^	0/6	0/6	0/6

## Discussion

Breast CSCs, like all other CSCs, have been suggested to contribute to tumor development and progression [17,18]. Thus, identification of breast CSCs is very important not only for understanding the mechanism of breast cancer but also providing novel strategies for its successful treatment. Previous studies have used specific molecular markers and ultrastructures to identify and isolate breast CSCs [19-22]. It is well known that the surface ultrastructure of cancer cells is unique and important for cancer development [23,24], since the surface ultrastructure of cells can predict cell-cell and cell-matrix contact, movability, and adhesion ability. However, to date, information regarding the cell surface ultrastructure of breast CSCs is limited. Herein, in this study, we analyzed the surface ultrastructure of different subsets of MCF7 cells after sorting by the known breast CSC markers CD44 and CD24. We found that the CD44^+^/CD24^-/low^ breast cancer cells showed numerous protrusions on the cell surface and had many microvilli and pseudopodia, indicating such ultrastructures may contribute to higher tumorigenesis and invasion ability. The association between surface marker and surface ultrastructure of CSCs is quite interesting, i.e., CSCs might have a special surface ultrastructure and provide valuable information to identify CSCs and biological significance. Further understanding of these features could help us to develop a novel clinical cancer-targeted therapy. However, at the current stage, due to lack of standard CSC surface ultrastructure data, it is difficult to quantify the CSCs pseudopodia. We will develop a methodology to quantify them in the near future.

The CD44 antigen, encoded by the *CD44* gene on chromosome 11, is a cell-surface glycoprotein involved in cell–cell interactions, cell adhesion, and migration [25]. CD44 has been reported as a cell surface marker for some breast and prostate CSCs, but it is associated with an increased survival duration of epithelial ovarian cancer patients [5,26]. Likewise, CD24 is encoded by the *CD24* gene and is also a cell adhesion molecule [27]. The expression of CD24 is associated with tumor development and plays a critical role in various cancer metastases [28,29]. However, more recently, researchers in the field of CSCs have used these two proteins as markers to identify breast CSCs with high CD44 expression and negative or low CD24 expression [2,3]. In this study, we also sorted breast cancer MCF7 cells using these two markers to obtain four subpopulations of MCF7 cells. Consistent with previous studies, we found that the CD44^+^/CD24^-/low^ population had high tumorigenic potential and invasion capacity.

Because *in vivo* tumor formation is the gold standard for identification of CSCs [30], our current data further confirmed that CD44^+^/CD24^-^ cells have the highest tumorigenic capacity compared to the other three subsets of MCF7 cells; however, the tumorigenic capacity of CD44^+^/CD24^-^ cells was not as strong as that found in a previous report [2]. In our current study, only one of six mice formed a breast cancer xenograft with 1×10^3^ cells/injection. However, our current data also showed that CD44^+^/CD24^+^ cells formed tumor xenografts in two of six mice after the injection of 1×10^5^ cells. This finding indicates that CD24 is not as good as CD44 as a marker for the identification of breast CSCs. We further analyzed the tumor weight and size in different groups; however, we did not observe any significant differences among them, indicating that CSCs might contribute to cancer initiation but not tumor progression.

## Conclusion

In summary, this study systematically investigated the ability of CD44 and CD24 proteins as markers to identify breast CSCs among MCF7 cells using flow cytometry to sort the MCF7 cells with these two proteins into four subsets. Moreoever, CD44^+^/CD24^-^ cells showed a unique surface ultrastructure by SEM and had the highest tumorigenic potential in tumor cell invasion and nude mouse xenograft assays. Further studies will determine whether CD44 directly contributes to the unique surface ultrastructure and tumorigenic capacity.

## Abbreviations

CSCs: Cancer stem cells; SEM: Scanning electron microscopy; DMEM: Dulbecco’s modified eagle’s medium; SCID: Severe combined immunodeficient; FCS: Fetal calf serum; PBS: Phosphate-buffered saline.

## Competing interest

The authors declare that they have no competing interest.

## Authors’ contributions

WH, YC, YY, and TW designed research; WH, YC, HZ, and TW performed research; WH, YC, YY, and HZ analyzed data; WH, and YW wrote the paper. All authors read and approved the final manuscript.

## References

[B1] Al-Hajj M Becker MW Wicha M Weissman I Clarke MF Therapeutic implications of cancer stem cells Curr Opin Genet Dev 2004 14 43 47 10.1016/j.gde.2003.11.007 15108804

[B2] Al-Hajj M Wicha MS Benito-Hernandez A Morrison SJ Clarke MF Prospective identification of tumorigenic breast cancer cells Proc Natl Acad Sci USA 2003 100 3983 3988 10.1073/pnas.0530291100 12629218 PMC153034

[B3] Charafe-Jauffret E Ginestier C Iovino F Wicinski J Cervera N Finetti P Hur MH Diebel ME Monville F Dutcher J Brown M Viens P Xerri L Bertucci F Stassi G Dontu G Birnbaum D Wicha MS Breast cancer cell lines contain functional cancer stem cells with metastatic capacity and a distinct molecular signature Cancer Res 2009 69 1302 1313 10.1158/0008-5472.CAN-08-2741 19190339 PMC2819227

[B4] Li F Tiede B Kang Y Massagué J Beyond tumorigenesis: cancer stem cells in metastasis Cell Res 2007 17 3 14 10.1038/sj.cr.7310118 17179981

[B5] Velasco-Velázquez MA Popov VM Lisanti MP Pestell RG The role of breast cancer stem cells in metastasis and therapeutic implications Am J Pathol 2011 179 2 11 10.1016/j.ajpath.2011.03.005 21640330 PMC3123864

[B6] Dean M Fojo T Bates S Tumour stem cells and drug resistance Nat Rev Cancer 2005 5 275 284 10.1038/nrc1590 15803154

[B7] Singh SK Clarke ID Terasaki M Bonn VE Hawkins C Squire J Dirks PB Identification of a cancer stem cell in human brain tumors Cancer Res 2003 63 5821 5828 14522905

[B8] O'Brien CA Pollett A Gallinger S Dick JE A human colon cancer cell capable of initiating tumour growth in immunodeficient mice Nature 2007 445 106 110 10.1038/nature05372 17122772

[B9] Zhang S Balch C Chan MW Lai HC Matei D Schilder JM Yan PS Huang TH Nephew KP Identification and characterization of ovarian cancer-initiating cells from primary human tumors Cancer Res 2008 68 4311 4320 10.1158/0008-5472.CAN-08-0364 18519691 PMC2553722

[B10] Tsuchiya S Li F Electron microscopic findings for diagnosis of breast lesions Med Mol Morphol 2005 38 216 224 10.1007/s00795-005-0300-9 16378230

[B11] Zhao YD Huang Q Zhang TY Dong J Wang AD Ding F Lan Q Gu XS Qin ZH [Ultrastructural analysis of glioma stem cells-progenitors] Zhonghua Zhong Liu Za Zhi 2008 30 663 667 19173906

[B12] Paniz Mondolfi AE Slova D Fan W Attiyeh FF Afthinos J Reidy J Pang Y Theise ND Mixed adenoneuroendocrine carcinoma (MANEC) of the gallbladder: a possible stem cell tumor? Pathol Int 2011 61 608 614 10.1111/j.1440-1827.2011.02709.x 21951672

[B13] Pham PV Phan NL Nguyen NT Truong NH Duong TT Le DV Truong KD Phan NK Differentiation of breast cancer stem cells by knockdown of CD44: promising differentiation therapy J Transl Med 2011 9 209 10.1186/1479-5876-9-209 22152097 PMC3251542

[B14] Zielske SP Spalding AC Wicha MS Lawrence TS Ablation of breast cancer stem cells with radiation Transl Oncol 2011 4 227 233 21804918 10.1593/tlo.10247 PMC3140010

[B15] Shipitsin M Campbell LL Argani P Weremowicz S Bloushtain-Qimron N Yao J Nikolskaya T Serebryiskaya T Beroukhim R Hu M Halushka MK Sukumar S Parker LM Anderson KS Harris LN Garber JE Richardson AL Schnitt SJ Nikolsky Y Gelman RS Polyak K Molecular definition of breast tumor heterogeneity Cancer Cell 2007 11 259 273 10.1016/j.ccr.2007.01.013 17349583

[B16] Abraham BK Fritz P McClellan M Hauptvogel P Athelogou M Brauch H Prevalence of CD44+/CD24-/low cells in breast cancer may not be associated with clinical outcome but may favor distant metastasis Clin Cancer Res 2005 11 1154 1159 15709183

[B17] Wicha MS Cancer stem cell heterogeneity in hereditary breast cancer Breast Cancer Res 2008 10 105 10.1186/bcr1990 18423071 PMC2397530

[B18] Park SY Lee HE Li H Shipitsin M Gelman R Polyak K Heterogeneity for stem cell-related markers according to tumor subtype and histologic stage in breast cancer Clin Cancer Res 2010 16 876 887 10.1158/1078-0432.CCR-09-1532 20103682 PMC2818503

[B19] Ginestier C Hur MH Charafe-Jauffret E Monville F Dutcher J Brown M Jacquemier J Viens P Kleer CG Liu S Schott A Hayes D Birnbaum D Wicha MS Dontu G ALDH1 is a marker of normal and malignant human mammary stem cells and a predictor of poor clinical outcome Cell Stem Cell 2007 1 555 567 10.1016/j.stem.2007.08.014 18371393 PMC2423808

[B20] Smith GH Chepko G Mammary epithelial stem cells Microsc Res Tech 2001 52 190 203 10.1002/1097-0029(20010115)52:2<190::AID-JEMT1005>3.0.CO;2-O 11169867

[B21] Chepko G Dickson RB Ultrastructure of the putative stem cell niche in rat mammary epithelium Tissue Cell 2003 35 83 93 10.1016/S0040-8166(02)00107-6 12747930

[B22] Pierce GB Nakane PK Martinez-Hernandez A Ward JM Ultrastructural comparison of differentiation of stem cells of murine adenocarcinomas of colon and breast with their normal counterparts J Natl Cancer Inst 1977 58 1329 1345 857028 10.1093/jnci/58.5.1329

[B23] Black PH Shedding from the cell surface of normal and cancer cells Adv Cancer Res 1980 32 75 199 7008543 10.1016/s0065-230x(08)60361-9

[B24] Nicolson GL Poste G The cancer cell: dynamic aspects and modifications in cell-surface organization (first of two parts) N Engl J Med 1976 295 197 203 10.1056/NEJM197607222950405 775336

[B25] Spring FA Dalchau R Daniels GL Mallinson G Judson PA Parsons SF Fabre JW Anstee DJ The Ina and Inb blood group antigens are located on a glycoprotein of 80,000 MW (the CDw44 glycoprotein) whose expression is influenced by the In(Lu) gene Immunology 1988 64 37 43 2454887 PMC1385183

[B26] Sillanpää S Anttila MA Voutilainen K Tammi RH Tammi MI Saarikoski SV Kosma VM CD44 expression indicates favorable prognosis in epithelial ovarian cancer Clin Cancer Res 2003 9 5318 5324 14614016

[B27] Li D Zheng L Jin L Zhou Y Li H Fu J Shi M Du P Wang L Wu H Chen GY Zheng P Liu Y Wang FS Wang S CD24 polymorphisms affect risk and progression of chronic hepatitis B virus infection Hepatology 2009 50 735 742 10.1002/hep.23047 19610054

[B28] Nestl A Von Stein OD Zatloukal K Thies WG Herrlich P Hofmann M Sleeman JP Gene expression patterns associated with the metastatic phenotype in rodent and human tumors Cancer Res 2001 61 1569 1577 11245467

[B29] Aigner S Ruppert M Hubbe M Sammar M Sthoeger Z Butcher EC Vestweber D Altevogt P Heat stable antigen (mouse CD24) supports myeloid cell binding to endothelial and platelet P-selectin Int Immunol 1995 7 1557 1565 10.1093/intimm/7.10.1557 8562500

[B30] Magee JA Piskounova E Morrison SJ Cancer stem cells: impact, heterogeneity, and uncertainty Cancer Cell 2012 21 283 296 10.1016/j.ccr.2012.03.003 22439924 PMC4504432

